# Codon Usage Bias in Autophagy-Related Gene 13 in Eukaryotes: Uncovering the Genetic Divergence by the Interplay Between Nucleotides and Codon Usages

**DOI:** 10.3389/fcimb.2021.771010

**Published:** 2021-11-05

**Authors:** Yicong Li, Rui Wang, Huihui Wang, Feiyang Pu, Xili Feng, Li Jin, Zhongren Ma, Xiao-xia Ma

**Affiliations:** ^1^ Biomedical Research Center, Northwest Minzu University, Lanzhou, China; ^2^ Viterbi School of Engineering, University of Southern California, Los Angeles, CA, United States

**Keywords:** autophagy-related gene 13, phylogenetic analyses, nucleotide usage, synonymous codon usage, nucleotide composition distribution

## Abstract

Synonymous codon usage bias is a universal characteristic of genomes across various organisms. Autophagy-related gene 13 (*atg13)* is one essential gene for autophagy initiation, yet the evolutionary trends of the *atg13* gene at the usages of nucleotide and synonymous codon remains unexplored. According to phylogenetic analyses for the *atg13* gene of 226 eukaryotic organisms at the nucleotide and amino acid levels, it is clear that their nucleotide usages exhibit more genetic information than their amino acid usages. Specifically, the overall nucleotide usage bias quantified by information entropy reflected that the usage biases at the first and second codon positions were stronger than those at the third position of the *atg13* genes. Furthermore, the bias level of nucleotide ‘G’ usage is highest, while that of nucleotide ‘C’ usage is lowest in the *atg13* genes. On top of that, genetic features represented by synonymous codon usage exhibits a species-specific pattern on the evolution of the *atg13* genes to some extent. Interestingly, the codon usages of atg13 genes in the ancestor animals (*Latimeria chalumnae*, *Petromyzon marinus, and Rhinatrema bivittatum*) are strongly influenced by mutation pressure from nucleotide composition constraint. However, the distributions of nucleotide composition at different codon positions in the *atg13* gene display that natural selection still dominates *atg13* codon usages during organisms’ evolution.

## Introduction

Autophagy (or macroautophagy) is an intracellular protein-degradation process that is conserved from yeast to mammalian cells. This self-degradation system, where lysosomes/vacuoles degrade unnecessary cytoplasmic components from nutrient starvation, was first discovered in mammalian cells in the 1960s (Ashford and Porter, 1962). With the ongoing studies on autophagy in yeasts, a series of autophagy-related genes (*atg*) encoding proteins that are involved in autophagy membranes biogenesis came into research attention (Tsukada and Ohsumi, 1993; Thumm et al., 1994; Harding et al., 1995). By investigating the molecular machinery of autophagy, 35 different *atg* genes have been identified in the morphologic and biochemical pathways from yeast genetic studies. Among these autophagy-related genes, *atg1-10*, *atg12-14*, *atg16-18*, *atg29*, and *atg31* play important roles in shaping canonical autophagosomes (Nakatogawa et al., 2009). The identified *atg* genes are highly conserved across various eukaryotes. The findings of these proteins take the studies of autophagosome formation to the molecular level where the genetic characteristics of autophagy are further investigated in mammalian cells. While it has been believed that ATG proteins are mainly involved in autophagy, some of them were actually identified to display non-autophagic functions, such as antiviral responses, cell proliferation, and development assistance (Kroemer and Levine, 2008; Radoshevich et al., 2010; Fan et al., 2017; Ma et al., 2020). Such diverse dynamics of the ATG proteins attract deeper investigations at a genetic level.

ATG13 is a serine-rich protein that contains several phosphorylation sites and other critical genetic regions (Fujioka et al., 2014). For example, ATG13-17BR and ATG13-17LR, two important functional regions, are able to interact with ATG17 (another autophagy-related protein) to construct a complex with ATG29 and ATG31 (Fujioka et al., 2014; Yamamoto et al., 2016). In addition, ATG13, which is the direct substrate of the target of rapamycin complex 1 (TORC1), is hyper-phosphorylated under nutrient-rich conditions (Funakoshi et al., 1997; Kamada et al., 2000; Kamada et al., 2010). In addition, another functional domain of ATG13, the MIM domain, can bind to the protein kinase ATG1 (Fujioka et al., 2014). The MIM domain under phosphorylation weakens the interaction between ATG1 and ATG13, therefore, as in the case of ATG17, the interplay between ATG1 and ATG13 can be facilitated by starvation in a TORC1-dependent regulation manner (Kamada et al., 2000; Fujioka et al., 2014; Noda, 2017).

The degeneracy of the genetic code results in the synonymous codon where different codons would translate into the same amino acid. The genetic codes that define the amino acid sequence of a protein are able to mediate the efficiency and rate of translation (Brule and Grayhack, 2017). Synonymous codon usages are commonly regarded as a bridge between nucleotide usages and amino acid compositions (Novoa and Ribas De Pouplana, 2012). Interestingly, the homogenous gene among different species may prefer different sets of synonymous codon usage, and the overall codon usage pattern appears to be similar among closely related species but displays divergence among distantly related species (De Mandal et al., 2020; Ge et al., 2020; Liu et al., 2020; Gupta et al., 2021). The reasons behind the uneven usage frequencies of synonymous codons in protein-coding have been puzzling molecular evolutionary researchers for decades (Pedrotti and Guerra, 1989). In animals, studies of the population genomics mark the codon usage bias by the effects of translational selection and GC-biased gene conversion on population transcriptomics data (Galtier et al., 2018). Moreover, the overall codon usage bias and the interplay between nucleotide usages and synonymous codon usage patterns manifest the non-randomness in coding sequences, making it an important area of study for cellular genetic processes (Fedorov et al., 2002; Carlini, 2005; Nabiyouni et al., 2013). In addition, synonymous codon usage bias also has strong correlations with the formations of different folding structures of protein, resulting in altering biological functions of protein (Komar et al., 1999; Zhou et al., 2009; Saunders and Deane, 2010; Weygand-Durasevic and Ibba, 2010; Zhou et al., 2013b). Having a large intrinsically disordered region that can tether ATG17 molecules and lead to supramolecular ATG1 complex assembly, ATG13 works as an essential autophagy factor in mediating the supramolecular assembly for autophagy initiation (Yamamoto et al., 2016). While the existing sequencing technology has granted more accessibility to the studies on eukaryotic genome patterns, the knowledge about the conservation and evolution of *atg* genes across eukaryotes still remains unclear. Among the questions which have been raised is: do *atg13* genes derived from different species display similar patterns in their genetic characterizations on synonymous codon usage or not? Here, in this study, we wanted to uncover more insights into how nucleotide, synonymous codon usage, and amino acid usages act on the *atg13* gene evolutionary trend in eukaryotes by investigating the *atg13* genes derived from 226 different eukaryotic organisms.

## Materials and Methods

### Phylogenetic Analyses for Different *atg13* Genes at the Nucleotide and Amino Acid Levels

The different *atg13* genes derived from 226 eukaryotic organisms were obtained from the National Center for Biotechnology (NCBI) Genome database (https://www.ncbi.nlm.nih.gov/) (**Table S1**). According to the whole coding sequences of 226 *atg13* genes (**Table S1**), the two phylogenetic trees were constructed by Maximum Composite Likelihood model with UPGMA statistical method at the nucleotide and amino acid levels, respectively.

### Nucleotide Usage Bias of the *atg13* Genes

To quantify the degree of the bias at the overall nucleotide usage of every nucleotide position in a code, the information entropy formula was used for calculating the nucleotide usage bias of the *atg13* gene per species.


$$Entropy=-\sum_{i}f_{i}\times \log_{2}(f_{i})$$

$$f_{i}=\frac{F_{i}}{F(A)+F(T)+F(G)+F(C)}$$

where *f_i_* stands for the probability of the specific nucleotide (*F_i_*), *F_i_* means the number of occurrences of the specific nucleotide. The value for information entropy of nucleotide usage bias reflects how dispersed the contribution of the overall usage of the four nucleotide types is: the higher the value, the more uniform the usage between different nucleotides is; in contrast, a lower value reflects a more biased usage between different nucleotides (Ge et al., 2020).

In addition, to assess the usage distribution for each nucleotide across the three positions in a code of *atg13* gene, we also implemented the strategy of information entropy to quantify the overall usage bias for each nucleotide.


$$Entropy=-\sum_{i}f_{i}\times \log_{2}(f_{i})$$

$$f_{n}=\frac{F_{n}}{F(N_{1})+F(N_{2})+F(N_{3})}$$

where *f_n_* stands for the probability of the specific nucleotide type (‘*n*’ corresponding to A, T, C, or G), *F_n_* means the number of occurrences of the specific nucleotide type. *F(N_1_)*, *F(N_2_)*, and *F(N_3_)* correspond to the nucleotide content at the 1^st^, 2^nd^, and 3^rd^ codon positions, respectively.

### Relative Synonymous Codon Usage Calculation

The relative synonymous codon usage value (RSCU) was used to quantify the synonymous codon usage bias for each *atg13* gene in this study. In principle, three canonical stop codons (UAA, UAG, and UGA), AUG for Met, and UGG for Try are unavailable for RSCU analyses. According to the previous reports for estimating synonymous codon usage (Sharp and Li, 1986; Zhou et al., 2013a; Gun et al., 2018), the related standard was established that codons with RSCU values >1.6 would be regarded as overrepresented ones, while codons with RSCU values <0.6 were thought to be underrepresented. Therefore, a synonymous codon with an RSCU value <0.6 or >1.6 can be defined as a biased one.

To better quantify the dispersion of synonymous codon usages in *atg13* genes across different eukaryotic species, we introduced the formula (Vs=σx¯) into this study.


$$\sigma =\sqrt{\frac{\sum_{i=1}^{N}{\left(x_{i}-\bar{x}\right)}^{2}}{N-1}}$$

where {*x_1_*, *x_2_*, *x_3_*, …, *x_N_*} are the RSCU values of the given synonymous codon of *atg13* gene derived from the specific eukaryotic organisms in this study; $\bar{x}$ means the average value of these RSCU values; and ‘*N*’ is the number of eukaryotic species in this study. Based on *V_s_* values, the lower the *V_s_* value is, the more stable the specific synonymous codon usage is.

Genetic divergence at synonymous codon and amino acid usages for *atg13* gene:

To visualize the overall genetic divergence at synonymous codon or amino acid usages among the 226 *atg13* genes, we implemented principal component analysis (PCA), a multivariate statistical pathway. Specifically, the 59 canonical synonymous codons were regarded as a multi-dimensional vector (each dimension corresponds to the RSCU value for each sense codon). Similarly, to estimate ATG13 protein evolution across these eukaryotic organisms, the 20 canonical amino acids were regarded as a 20-dimensional vector in regard to the different frequencies of the amino acid.

### Analyses for the Overall Codon Usage Patterns of *atg13* Gene Influenced by Selective Forces:

To quantify the absolute magnitude of bias at the overall codon usage for each *atg13* gene, the effective number of codons (ENC) calculation was carried out, ranking the codon usage bias for *atg13* gene per eukaryotic organism in this study. While ENC values can range from 20 to 61 (Wright, 1990), the lower the value is, the more biased the absolute codon usage is. It has been pointed out that one gene would have a significant codon bias when the ENC value is less than 35 (Comeron and Aguadé, 1998). To further evaluate the interplay between mutation pressure and natural selection forcing evolutionary trends of *atg13* gene at codon usages, the parity rule 2 (PR2) plot analysis was performed for each sample in this study. In detail, the PR2 plot is constructed by the ordinate (AU-bias [A_3_/(A_3_+T_3_)] and the abscissa (the GC-bias [G_3_/(G_3_+C_3_)] (Butt et al., 2016). Here, A_3_, T_3_, G_3,_ and C_3_ stand for frequencies of occurrence of the corresponding base at the third codon position of the four-codon amino acids of gene population). As for the plot represented by AU-bias vs. GC-bias, the center of the plot, where both coordinate are 0.5, is where base A equal to base U and base G equal to base C (PR2), with no bias between the influence of the mutation and selection rates (substitution rates) (Sueoka, 1995; Sueoka, 1999).

### Estimating Effects of Nucleotide Compositional Distribution of *Atg13* Gene on Its Protein Property

For a better understanding of the compositional distribution influenced by nucleotide usages in these *atg13* genes, we implemented nucleotide skew variants. From the variants, we would be able to explore the previously hidden relationships between the overall nucleotide skew across coding sequence and nucleotide composition distribution in the specific position in a code. AT skew and GC skew were commonly used for displaying the compositional distribution of nucleotide usages (Tillier and Collins, 2000). Positive AT and GC skew values represent a higher proportion of A base over T and similarly, higher G over C and vice versa. The deviation of skew value from zero marks the disproportional usage of two bases in the gene. Based on the analysis strategy of GC and AT skews, we also calculated purine skew [(A-G)/(A+G)], pyrimidine skew [(T-C)/(T+C)], keto skew [(T-G)/(T+G)] and amino askew [(A-C)/(A+C)]. The bivariate correlation analyses were conducted between nucleotide skew data for the specific codon position and the corresponding overall nucleotide skew data from using the Pearson method with a two-tailed test.

## Results

### The Obvious Specific-Species Genetic Diversity for *atg13* Genes

**Figure 1:**
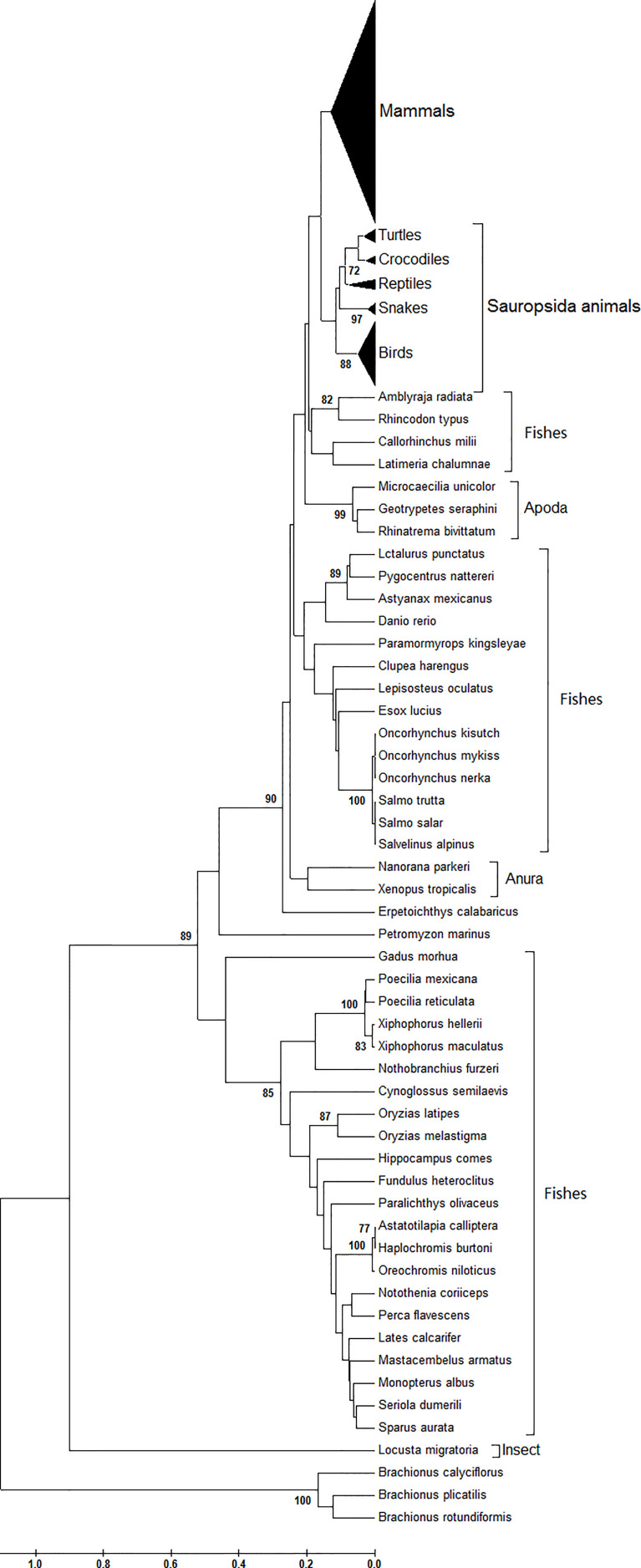
Phylogenetic analysis of *atg13* genes of 226 eukaryotic organisms. This phylogenetic tree was constructed by the Maximum Composite Likelihood model with UPGMA statistical method at the nucleotide level. Branches leading to these groups of sequences were supported by 100% of bootstrap replications in all cases.

Serving as one conserved gene across eukaryotic species, the phylogenetic tree of the *atg13* gene displays the obvious species-specific genetic divergences at the nucleotide level (**Figure 1**). As shown in **Figure 1**, the three *Brachionus* spp. (*B. calyciflorus*, *B. plicatilis*, and *B. rotundiformis*) have a significantly divergent evolutionary trend relative to those of other animals. Likewise, *Locusta migratoria* (a kind of insect) retains its specific evolutionary trend at the nucleotide level. As for the fish family in this study, it can be observed that there are three significantly different genetic branches. Specifically, the three divergent genetic populations do not correlate to geographic factors or living habits on the *atg13* evolutionary trends of fishes. However, the species-specific nature still plays an important role in *atg13* genes among different kinds of fishes. Turning to *atg13* genes in the amphibian family in this study, Anura (*Nanorana parkeri* and *Xenopus tropicalis*) does not serve as a sister group with Apoda (*Microcaecilia unicolor*, *Geotrypetes seraphini*, or *Rhinatrema bivittatum*). Interestingly, *Petromyzon marinus* and *Erpetoichthys calabaricus* (two ancient animals) exhibit similar genetic niches of *atg13* gene at nucleotide organization. Likewise, the species-specific evolutionary dynamic also acts on genetic divergence of *atg13* genes between Mammals and *Sauropsida* animals (**Figure 1**).

**Figure 2:**
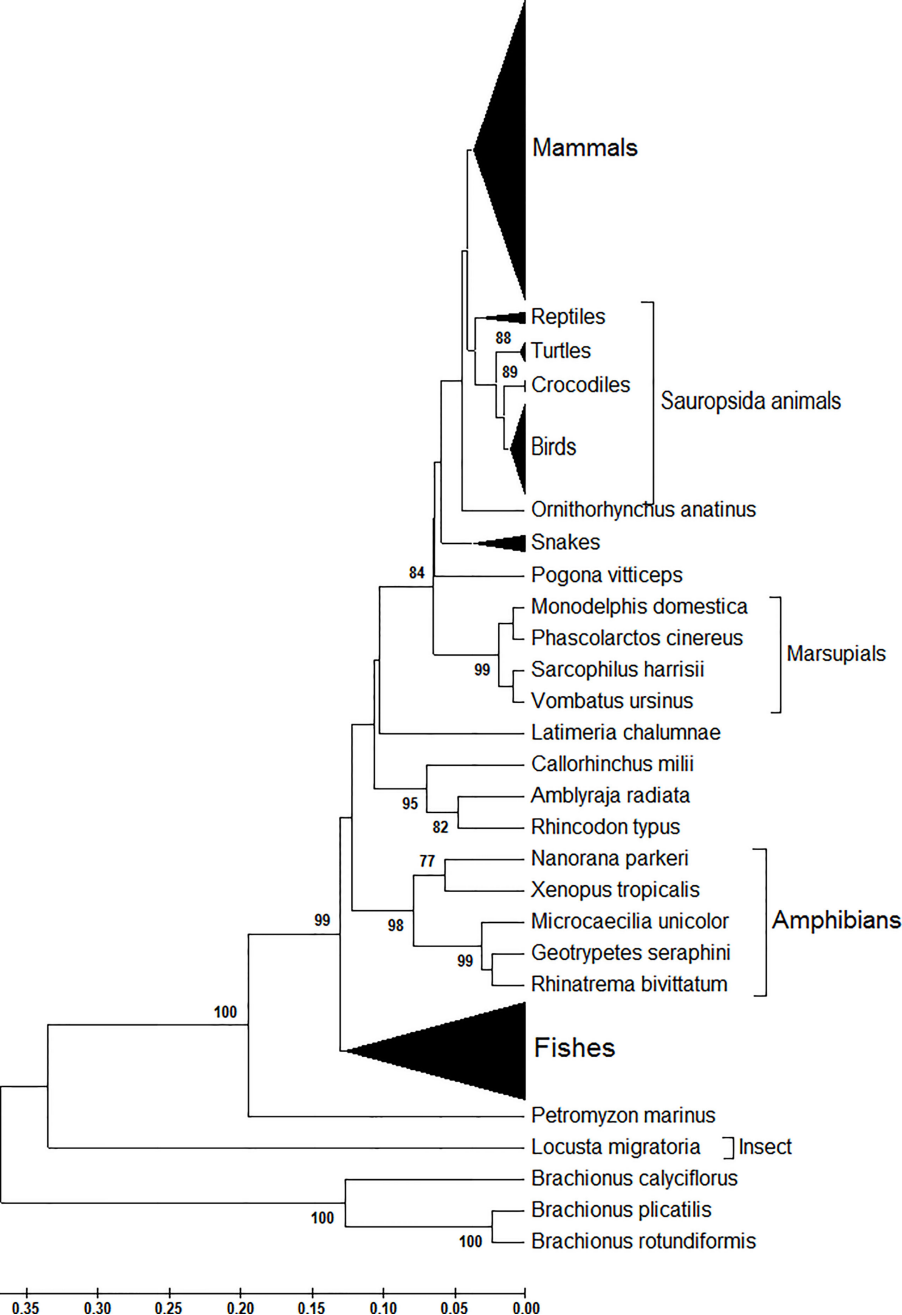
Phylogenetic analysis of ATG13 protein sequences of 226 eukaryotic organisms. This phylogenetic tree was constructed by the Maximum Composite Likelihood model with UPGMA statistical method at the nucleotide level. Branches leading to these groups of sequences were supported by 100% of bootstrap replications in all cases.

Next, from the phylogenetic tree, we found that at the amino acid level, *atg13* genes display a species-specific dynamic in their evolutionary trends (**Figure 2**). Compared with the genetic diversity of *atg13* genes with other eukaryotes (**Figure 1**), ATG13 proteins of both *Brachionus* spp. and *L. migratoria* still display distinct evolutionary trends in regard to their species, while the majority of the fish in this study has been classified into one genetic group. As for the genetic diversity of ATG13 proteins, we found that Anura and Apoda compose of a sister group. In addition, the four marsupials (*Monodelphis domestica*, *Phascolarctos cinereus*, *Sarcophilus harrisii*, and *Vombatus ursinus*) and *Ornithorhynchus anatinus* are found to have different genetic niches in comparison to the rest mammals in this study (**Figure 2**). Taken together, the evolutionary trend of the *atg13* gene is strongly influenced by the species-specific selection at either nucleotide or amino acid l1vel to different degrees.

### Amino Acid Composition Constraint Influences Nucleotide Usages in the *atg13* Gene

**Figure 3:**
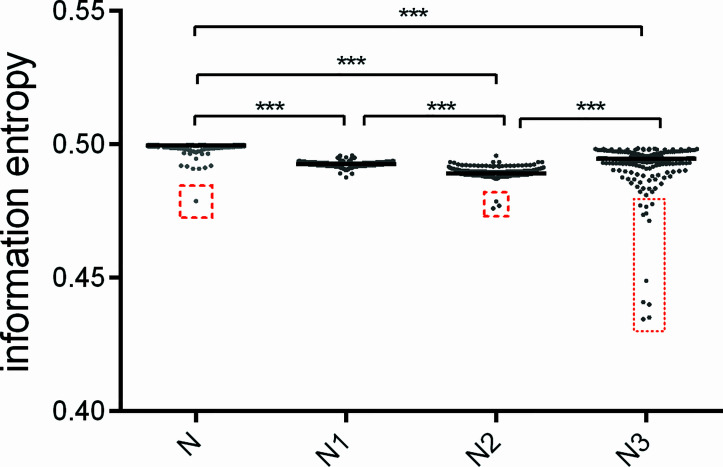
The patterns of nucleotide usage bias for *atg13* genes of 226 eukaryotic organisms are represented by information entropy. The ‘N’ means the overall nucleotide usage bias across the complete gene, The ‘N1’, ‘N2’, and ‘N3’ stand for the patterns of nucleotide usage bias at the first, second, and third codon positions, respectively. The red box with broken lines highlights the outliers which correspond to *atg13* genes of the specific organisms. *** means *p*-value < 0.001.

To quantify the magnitudes of nucleotide usage bias in different codon positions of these sequences, we used information entropy to estimate the differences of nucleotide usage bias in each position in comparison to the overall nucleotide usage bias for the whole coding sequence. Generally, the nucleotide usage biases in the first and second codon positions are significantly stronger than the overall usage bias (*p* value <0.001) (**Figure 3**), implying that nucleotide usage patterns for *atg13* genes are subject to selective forces derived from nucleotide composition constraint and translation selection. Of note, despite significantly different biases between the overall nucleotide usage bias and that of the third codon position, the observation of the highly variant ranks of nucleotide usage biases are carried out at the third codon position of *atg13* genes in some eukaryotic organisms (**Figure 3**). According to the data of nucleotide usage bias, the outliers are observed in columns ‘N’, ‘N2’, and ‘N3’. It can be found in **Figure 3** that *Brachionus calyciflorus* is included in column ‘N’, *Brachionus rotundiformis*, *Brachionus plicatilis*, and *Brachionus calyciflorus* in column ‘N2’, and *Gadus morhua*, *Oryzias latipes*, *Oryzias melastiqma*, *Brachionus calyciflrus*, *Petromyzon marinus*, *Fukomys damarensis*, *Nothobranchius furzeri*, *Poecilia reticulate*, *Poecilia Mexicana*, *Sarcophilus harrisii*, and *Fundulus heteroclitus* in column ‘N3’. These outliers suggest the existence of stronger selective forces acting on nucleotide usage patterns at the specific codon position.

**Figure 4:**
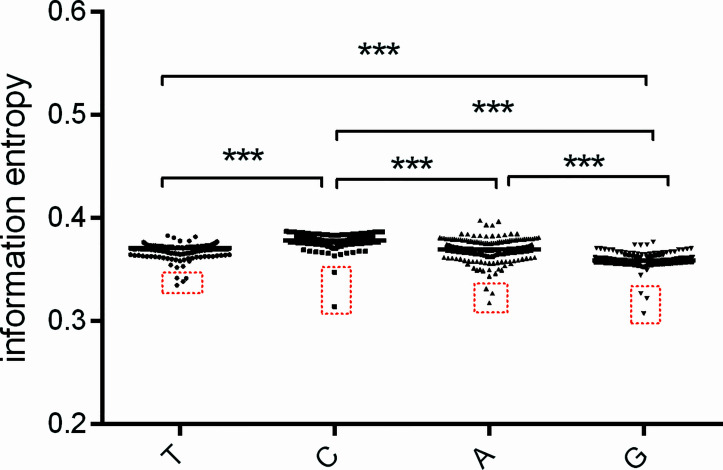
The usage bias for the specific nucleotide (A, T, G, or C) in the *atg13* genes of 226 eukaryotic organisms represented by information entropy. The red box with broken lines highlights the outliers which correspond to *atg13* genes of the specific organisms. *** means *p*-value < 0.001.

According to nucleotide usage bias at the three codon positions (**Figure 3**), we further estimated the usage bias extent of each type of nucleotide across all positions in a code for the *atg13* gene. The usage bias extent of nucleotide ‘G’ usage is highest, while that of nucleotide ‘C’ usage is lowest in *atg13* gene (**Figure 4**). Moreover, the outliers are also observed at the four nucleotide types, namely *Fundulus heteroclitus*, *Oryzias melastigma*, *Oryzias latipes*, and *Gadus morhua* in the column ‘T’; *Locusta migratoria* and *Brachionus calyciflorus* in the column ‘C’; *Oryzias latipes*, *Oryzias melastigma*, and *Fundulus heteroclitus* in the column ‘A’; *Brachionus plicatilis*, *Brachionus rotundiformis*, and *Brachionus calyciforus* in the column ‘G’. These outliers further prove that nucleotide usage bias for the *atg13* gene is more influenced by natural selection than by mutation pressure from the nucleotide composition constraint.

### Strong Selective Forces Acting On Synonymous Codon Usage Patterns of *atg13* Gene

Given the strong bias at nucleotide usages of the *atg13* gene (**Figures 3** and **4**), we subsequently calculated RSCU data for the *atg13* gene per species (**Table S2**). Generally, different eukaryotic organisms exhibit their specific codon usage patterns. Moreover, despite the higher composition of GC than AT in most of the genomes in this study, synonymous codons with neither G/C ends nor A/T end display a uniform pattern. To estimate the roles of nucleotide composition constraint and natural selection in synonymous codon usages, we estimated the dispersion extent (*V_s_* value) for each synonymous codon usage in *atg13* genes across these eukaryotic species. Among the synonymous codon usages (**Table 1**), some display highly variant usage patterns, including CTA for Leu (*V_s_* =0.718), TCG for Ser (*V_s_* =0.966), CCG for Pro (*V_s_* =0.903), GCG for Ala (*V_s_* =1.093), and CGC for Arg (*V_s_* =0.883). In the meantime, however, some exhibit strongly stable usage patterns, including TTT for Phe (*V_s_* =0.158), GTG for Val (*V_s_* =0.196), TCT for Ser (*V_s_* =0.188), CCT for Pro (*V_s_* =0.180), CAG for Gln (*V_s_* =0.159), AAG for Lys (*V_s_* =0.183), GAC for Asp (*V_s_* =0.162), and GAG for Glu (*V_s_* =0.197). These results prove the existence of the strong selective forces acting on synonymous codon usage patterns in the *atg13* gene with the specific species.

**Table 1:** The dispersion magnitude of synonymous codon usages for *atg13* genes across all species in this study.

	*Vs* value		*Vs* value		*Vs* value
TTT(F)	0.158	CCT(P)	0.180	AAA(K)	0.237
TTC(F)	0.430	CCC(P)	0.234	AAG(K)	0.183
TTA(L)	0.480	CCA(P)	0.290	GAT(D)	0.227
TTG(L)	0.339	CCG(P)	0.903	GAC(D)	0.162
CTT(L)	0.265	ACT(T)	0.250	GAA(E)	0.220
CTC(L)	0.267	ACC(T)	0.223	GAG(E)	0.197
CTA(L)	0.718	ACA(T)	0.339	TGT(C)	0.249
CTG(L)	0.242	ACG(T)	0.574	TGC(C)	0.544
ATT(I)	0.275	GCT(A)	0.203	CGT(R)	0.300
ATC(I)	0.304	GCC(A)	0.245	CGC(R)	0.883
ATA(I)	0.368	GCA(A)	0.337	CGA(R)	0.496
GTT(V)	0.244	GCG(A)	1.093	CGG(R)	0.571
GTC(V)	0.274	TAT(Y)	0.306	AGA(R)	0.351
GTA(V)	0.496	TAC(Y)	0.222	AGG(R)	0.264
GTG(V)	0.196	CAT(H)	0.296	GGT(G)	0.370
TCT(S)	0.188	CAC(H)	0.287	GGC(G)	0.206
TCC(S)	0.215	CAA(Q)	0.473	GGA(G)	0.303
TCA(S)	0.293	CAG(Q)	0.159	GGG(G)	0.286
TCG(S)	0.966	AAT(N)	0.266		
AGT(S)	0.428	AAC(N)	0.244		
AGC(S)	0.206				

From the variant RSCU data for these *atg13* genes (**Table S2**), 57 out of 59 synonymous codon usage patterns display a wide spectrum of RSCU values among different species. However, the synonymous codons (GCG for Ala and CTA for Leu) are suppressive selections (RSCU <1.0), except *atg13* genes of *Petromy marinus* (RSCU_GCG_=1.04), *Meleagris gallopavo* (RSCU_CTA_=1.02), *Numida meleagris* (RSCU_=CTA_=1.07), and *Cygnus atratus* (RSCU_CTA_=1.26). Interestingly, some synonymous codons are absent from *atg13* genes in some eukaryotic organisms, such as TTA, CTA & CTG for Leu; ATT & ATA for Ile; GTC & GTA for Val; TCG for Ser, CCG for Pro; ACG for Thr, GCG for Ala; TAT for Tyr; TGT & TGC for Cys, and CGC, CGA, CGG & AGA for Arg (**Table 2**), suggesting that not only do invertebrates with low rank (such as wheel animalcule) but also vertebrates with a high rank (such as *Homo sapiens, Pan troglodytes* and *Macaca mulatta*) would skip the synonymous codon selection. However, such a pattern has only been found in *atg13* genes. Taken together, it can be proven that the synonymous codon usage bias of *atg13* genes in different species is more prone to the natural selection power than to the nucleotide composition constraint.

**Table 2:** Some synonymous codons are absent from atg13 genes from different species.

Synonymous codon/Amino acid	Species
UUA(L)	*Callorhinchus milii* and *Chelonia mydas*
CUA(L)	*Astatotilapia calliptera, Callithrix jacchus, Castor Canadensis, Cercocebus atys, Chlorocebus sabaeus, Colobus angolensis palliates, Cricetulus griseus, Elephantulus edwardii, Fundulus heteroclitus, Gadus morhua, Gorilla gorilla, Grammomys surdaster, Homo sapiens, Hylobates moloch, Macaca fascicularis, Macaca mulatta, Macaca nemestrina, Mandrillus leucophaeus, Mesocricetus auratus, Mus caroli, Mus musculus, Nomascus leucogenys, Oreochromis niloticus, Pan paniscus, Pan troglodytes, Papio Anubis, Peromyscus leucopus, Piliocolobus tephrosceles, Pongo abelii, Rattus norvegicus, Rattus rattus, Rhinopithecus bieti, Rhinopithecus roxellana* and *Theropithecus gelada*
CUG(L)	*Brachionus calyciflorus*
AUU(I)	*Nothobranchius furzeri*
AUA(I)	*Petromyzon marinus*
GUC(V)	*Locusta migratoria*
GUA(V)	*Oryzias latipes*
UCG(S)	*Alligator mississippiensis, Alligator sinensis, Callithrix jacchus, Calypte anna, Castor canadensis, Cercocebus atys, Chlorocebus sabaeus, Colobus angolensis palliates, Crocodylus porosus, Dipodomys ordii, Elephantulus edwardii, Felis catus, Galeopterus variegates, Gavialis gangeticus, Gorilla gorilla, Homo sapiens, Hylobates moloch, Lctalurus punctatus, Macaca fascicularis, Macaca mulatta, Macaca nemestrina, Mandrillus leucophaeus, Monopterus albus, Mus musculus, Nomascus leucogenys, Notechis scutatus, Pan paniscus, Pan troglodytes, Pantherophis guttatus, Papio Anubis, Piliocolobus tephrosceles, Pongo abelii, Pseudonaja textilis, Python bivittatus, Rattus norvegicus, Rhincodon typus, Rhinopithecus bieti, Rhinopithecus roxellana, Thamnophis elegans* and *Theropithecus gelada*
CCG(P)	*Alligator mississippiensis, Alligator sinensis, Amblyraja radiate, Anolis carolinensis, Aquila chrysaetos chrysaetos, Calypte anna, Carlito syrichta, Castor Canadensis, Columba livia, Corvus brachyrhynchos, Corvus cornix cornix, Crocodylus porosus, Dipodomys ordii, Dromaius, Egretta garzetta, Falco peregrinus, Gavialis gangeticus, Geospiza fortis, Haliaeetus albicilla, Meleagris gallopavo, Melopsittacus undulates, Myotis lucifugus, Numida meleagris, Opisthocomus hoazin, Parus major, Pelodiscus sinensis, Phasianus colchicus, Pogona vitticeps, Python bivittatus, Rhincodon typus, Serinus canaria, Strigops habroptila, Sturnus vulgaris, Trichechus manatus latirostris, Tyto alba* and *Zootoca vivipara*
ACG(T)	*Danio rerio, Microcaecilia unicolor, Monopterus albus, Pantherophis guttatus, Protobothrops mucrosquamatus, Pseudonaja textilis, Python bivittatus* and *Xenopus tropicalis*
GCG(A)	*Ailuropoda melanoleuca, Aptenodytes forsteri, Calypte anna, Canis lupus dingo, Canis lupus familiaris, Capra hircus, Carlito syrichta, Castor canadensis, Chaetura pelagica, Chelonia mydas, Chelonoidis abingdonii, Chrysemys picta bellii, Colobus angolensis palliates, Corvus brachyrhynchos, Corvus cornix cornix, Dasypus novemcinctus, Delphinapterus leucas, Dromaius, Eumetopias jubatus, Falco peregrinus, Galeopterus variegates, Gallus gallus, Geospiza fortis, Gopherus evgoodei, Gorilla gorilla, Jaculus jaculus, Miniopterus natalensis, Monodelphis domestica, Myotis brandtii, Myotis davidii, Myotis lucifugus, Nannospalax galili, Nipponia Nippon, Notechis scutatus, Odobenus rosmarus, Opisthocomus hoazin, Pantherophis guttatus, Parus major, Pelecanus crispus, Phasianus colchicus, Podarcis muralis, Pogona vitticeps, Pseudonaja textilis, Pteropus alecto, Pteropus vampyrus, Python bivittatus, Rhincodon typus, Rousettus aegyptiacus, Serinus canaria, Sturnus vulgaris, Sus scrofa, Terrapene carolina triunguis, Thamnophis elegans, Trachemys scripta elegans, Trichechus manatus latirostris, Tupaia chinensis, Ursus arctos horribilis, Ursus maritimus, Zalophus californianus* and *Zootoca vivipara*
UAU(Y)	*Chelonia mydas* and *Oryzias latipes*
UGU(C)	*Protobothrops mucrosquamatus*
UGC(C)	*Halichoerus grypus, Hipposideros armiger, Mirounga leonina, Rhincodon typus* and *Rhinolophus ferrumequinum*
CGC(R)	*Alligator mississippiensis, Alligator sinensis, Amblyraja radiate, Anas platyrhynchos, Anolis carolinensis, Aptenodytes forsteri, Aythya fuligula, Bos Taurus, Calypte anna, Chaetura pelagica, Columba livia, Cygnus atratus, Dromaius, Egretta garzetta, Fulmarus glacialis, Gavia stellata, Geotrypetes seraphini, Haliaeetus albicilla, Latimeria chalumnae, Locusta migratoria, Manis javanica, Melopsittacus undulates, Microcaecilia unicolor, Nipponia Nippon, Oxyura jamaicensis, Paramormyrops kingsleyae, Pelecanus crispus, Podarcis muralis, Pogona vitticeps, Protobothrops mucrosquamatus, Pygoscelis adeliae, Rattus norvegicus, Rhinatrema bivittatum, Rhincodon typus, Strigops habroptila, Struthio camelus australis, Sturnus vulgaris, Tyto alba* and *Zootoca vivipara*
CGA(R)	*Fundulus heteroclitus, Gorilla gorilla, Loxodonta africana, Notothenia coriiceps, Ornithorhynchus anatinus, Pogona vitticeps* and *Sparus aurata*
CGG(R)	*Gekko japonicus, Lates calcarifer, Lepisosteus oculatus, Oncorhynchus kisutch, Oncorhynchus mykiss, Oncorhynchus nerka, Paralichthys olivaceus, Protobothrops mucrosquamatus, Rhinatrema bivittatum, Salmo salar, Salmo trutta* and *Salvelinus alpinus*
AGA(R)	*Protobothrops mucrosquamatus*

### Codon Usages of *atg13* Gene Strongly Influenced by Natural Selection

**Figure 5:**
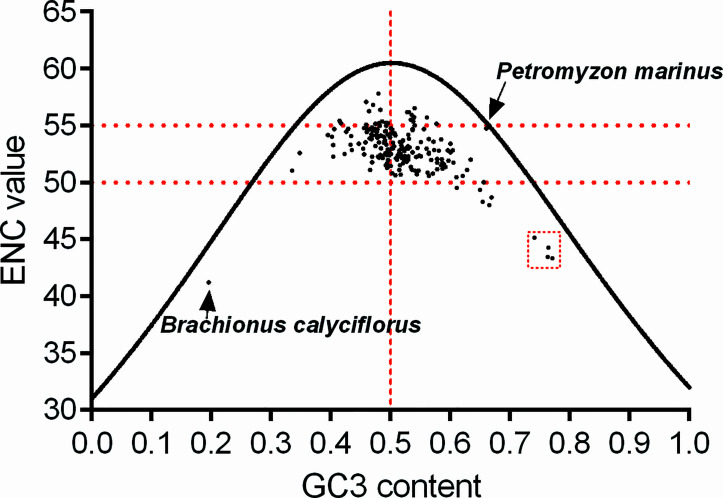
The relationship between ENC and GC content at the third synonymous codon position (GC3) in *atg13* genes of 226 eukaryotic organisms. The continuous curve line displays the expected codon usage if GC compositional constraints alone account for codon usage bias. The red broken lines highlight the distribution of *agt13* genes in this plot of ENC *vs.* GC3 content. In addition, the red box with broken lines highlights the outliers which correspond to *atg13* genes of the specific organisms.

Based on the synonymous codon usage patterns of *atg13* genes in different eukaryotic organisms (**Table S2**), we next estimated the magnitudes of the overall codon usage for transcript variants *via* ENC value vs. GC3 content. The plot of ENC value vs. GC3 content reveals that no eukaryotic organism owns significantly strong bias (ENC value < 35) at the overall codon usage for *atg13* gene, and most eukaryotic organisms own *atg13* genes with ENC values ranging from 50 to 55 (**Figure 5**). Specifically, these below-curve scattering dots prove that natural selection is stronger than the mutation pressure derived from nucleotide component constraint, especially *atg13* genes in *Brachionus calyciflorus, Gadus morhua, Oryzias melastigma, Oryzias latipes, and Fundulus heteroclitus*. Interestingly, with one exception for *Petromyzon marinus*, the nucleotide composition constraint dominates the mutation pressure in the overall codon usage formation of the *atg13* gene (**Figure 5**).

**Figure 6:**
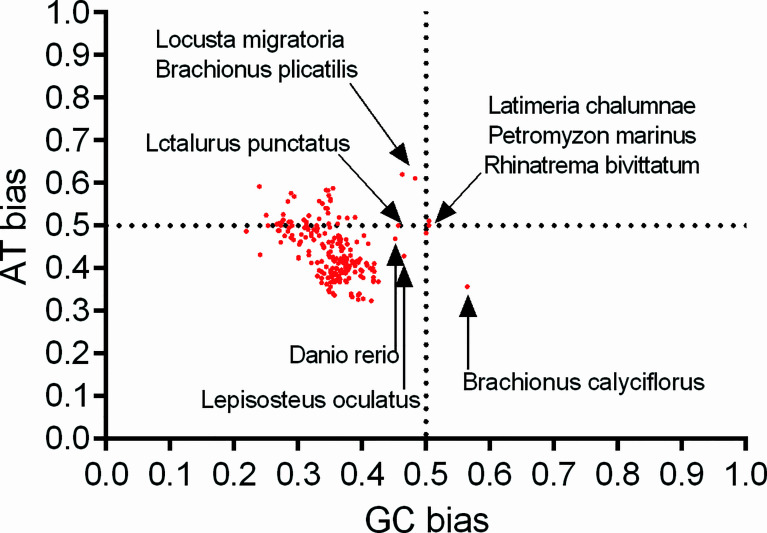
Parity rule 2 bias plot for *atg13* genes of 226 eukaryotic organisms.

Furthermore, to clarify whether the overall codon usage pattern of the *atg13* gene was shaped solely by natural selection, mutation pressure, or both, PR2 plot analysis was performed to evaluate the relationships between the nucleotides A-T contents and the G-C contents in the fourfold degenerate codon families (Ala, Gly, Pro, Thr, and Val). Specifically for *atg13* genes of these eukaryotic organisms, nucleotide C is selected more frequently than nucleotide G at the third codon position, except *Latimeria chalumnae* (GC bias=0.505) and *Brachionus calyciflorus* (GC bias=0.565) (**Figure 6**). Though there is a relatively big spectrum of AT bias for *atg13* genes of different species in this study, 37 out of 226 species have strong trends to select nucleotide A at the third codon position rather than nucleotide T (**Figure 6**). This unequal selection of different kinds of nucleotide at the third codon position exhibits the interplay between mutation pressure and natural selection on the overall codon usage in *atg13* genes for many eukaryotic organisms. In addition, the *atg13* genes of *Latimeria chalumnae, Petromyzon marinus, and Rhinatrema bivittatum* displayed almost even nucleotide usage (AT bias=0.5 and GC bias=0.5), and *Locusta migratoria, Brachionus plicatilis, Latalurus punctatus, Danio rerio*, and *Lepisosteus oculatus* are the next closest to the center than the rest of spices (**Figure 6**). These interesting genetic phenomena indicate that the *atg13* gene could store its evolutionary trace at altering synonymous codon usages during species evolutions.

### The Resembling Evolutionary Trends for *atg13* Genes in Mammals at Synonymous Codon Usages

**Figure 7:**
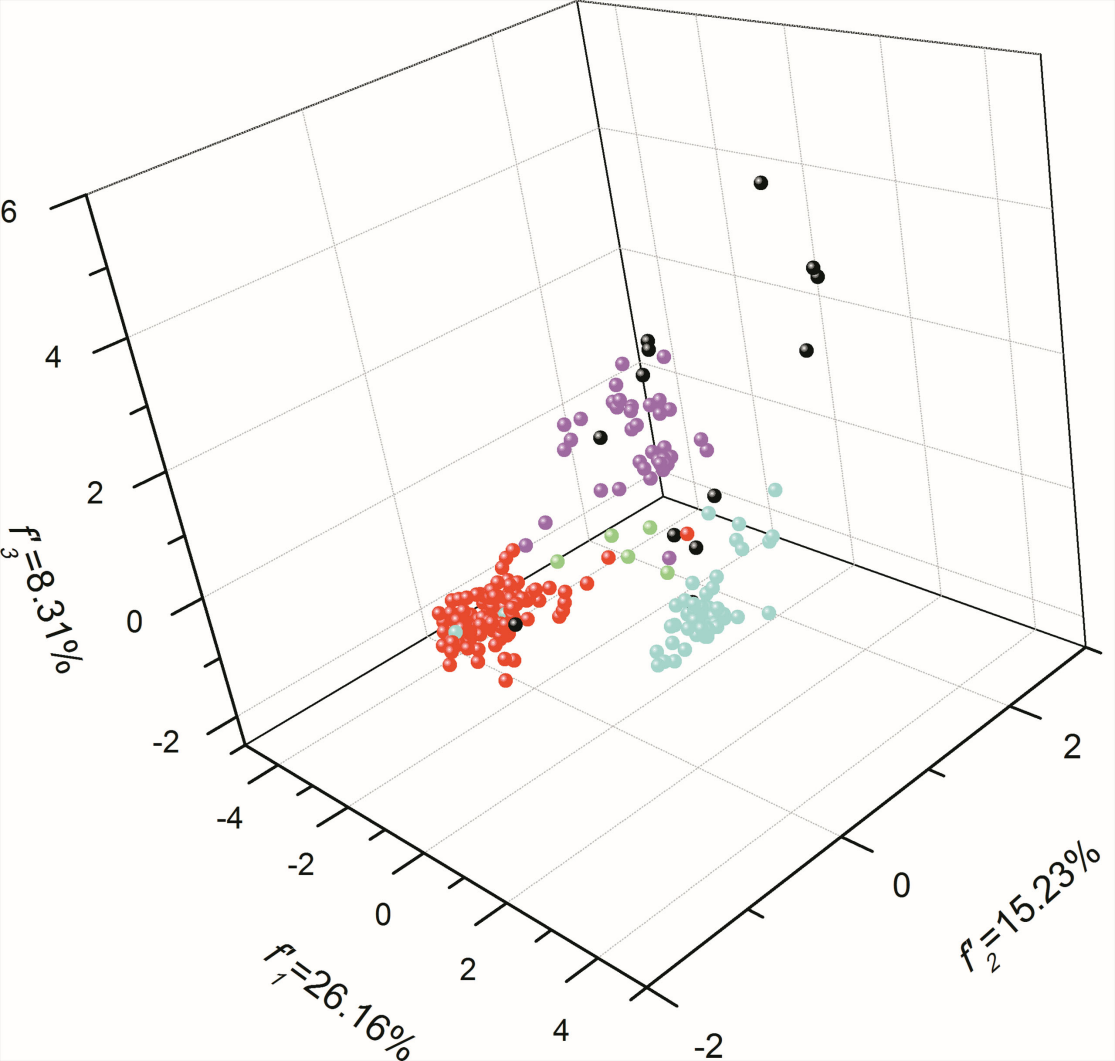
The plot for the overall codon usage visualized by PCA method for *atg13* genes of 226 eukaryotic organisms. The red dots mean mammals, the blue dots stand for *Sauropsida* animals, the purple dots mean fishes, the green dots mean amphibians, and the black dots correspond to the rest eukaryotic organisms in this study.

According to genetic characterizations reflected by synonymous codon usages (**Table S2**) and the overall codon usage bias (**Figure 5**), we next investigated the genetic diversity illustrated by 59 synonymous codon usages for these species in this study. Based on the classifications of mammals, *Sauropsida* animals, fishes, amphibians, and other organisms (the rest of the organisms in this study), the *atg13* genes of mammals and *Sauropsida* animals generally form two evolutionary groups, while *atg13* genes of amphibians appear to not form the resembling evolutionary trends (**Figure 7**). It can be clearly found that fish exhibit strong genetic dispersions at synonymous codon usages of the *atg13* gene. Moreover, *Brachionus calyciflorus, Brachionus plicatilis, and Brachionus rotundiformis* display the analogous evolutionary trends at synonymous codon usages of *atg13* gene, while *Locusta migratoria* exhibit its unique evolutionary trend relative to other species. The graphical representation of codon usage space for *atg13* genes of different species further indicates that selective forces involved with specific-species evolutionary dynamic play important roles in the genetic development of the *atg13* gene at the synonymous codon usage patterns.

### Nucleotide Composition Distributions at Different Positions in Codon Acting in the *atg13* Gene Context

Based on the selective forces acting on evolutionary trends of different *atg13* genes at codon usages (**Figures 5** and **6**), we wanted to investigate the roles of nucleotide composition distribution at the specific codon position in the *atg13* gene. It is unsurprising that conventional nucleotide composition distributions involved with GC3 and AT3 skews play more important roles than those of GC and AT skews at the first and second codon positions in influencing the selection of the GC and AT compositions in the *atg13* gene (**Table 3**). However, variations of GC and AT skews do not completely uncover the genetic variations around the *atg13* gene. Therefore, we evaluated the genetic effects on the distributions of purine (A & G) and pyrimidine (T & C) compositions at different codon positions on the *atg13* gene. We found similar effects from the purine and pyrimidine composition distributions on nucleotide usage patterns at the first and second codon positions of the *atg13* gene. However, distributions of purine composition and pyrimidine composition do not exhibit the same roles in nucleotide usages at the third codon position (**Table 3**). Meanwhile, we estimated the roles of keto (T & G) and amino (A & C) skews at different codon positions in the *atg13* gene. Interestingly, both usage variations of nucleotides (T & G) and nucleotides (A & C) play more important roles at the third codon position than at the first and second codon positions of the *atg13* gene. These results strongly indicate that while the transition caused by nucleotide substitutions mainly acts on the nucleotide usages at the first and second codon positions, transversion derived from nucleotide substitutions plays more important roles in the nucleotide usages at the third codon position of the *atg13* gene.

**Table 3:** The correlations between the overall nucleotide skew and the nucleotide skew at the specific codon position at the gene level.

	GC1 skew	GC2 skew	GC3 skew
**GC skew**	r=0.398***	r=0.448***	r=0.606***
	**AT1 skew**	**AT2 skew**	**AT3 skew**
**AT skew**	r=0.071**^NS^**	r=0.587***	r=0.888***
	**Purine1 skew**	**Purine2 skew**	**Purine3 skew**
**Purine skew**	r=0.862***	r=0.638***	r=0.939***
	**Pyrimidine1 skew**	**Pyrimidine2 skew**	**Pyrimidine3 skew**
**Pyrimidine skew**	r=0.859***	r=0.681***	r=-0.293***
	**Keto1 skew**	**Keto2 skew**	**Keto3 skew**
**Keto skew**	r=0.669***	r=0.618***	r=0.956***
	**Amino1 skew**	**Amino2 skew**	**Amino3 skew**
**Amino skew**	r=0.834***	r=0.559***	r=0.971***

***p < 0.001, ^NS^p > 0.05. NS, non-significance.

## Discussion

Synonymous codons are great representations of non-randomness in coding sequences, playing an important role in many different cellular processes. One of the most studied synonymous codon usages investigates the mRNA decay process and protein folding past translation. (Precup and Parker, 1987; Pop et al., 2014; Presnyak et al., 2015; Hia et al., 2019; Guimaraes et al., 2020). Autophagy, on the other hand, is one fundamental cellular process across eukaryotes, induced by various stimuli including environmental stressors which can be regarded as one selective force for cellular remodeling during organism development (Penaloza et al., 2006; Di Bartolomeo et al., 2010).

With the *atg13* gene being one essential factor for the formation of autophagy, its species-specific nucleotide usage in eukaryotic species has attracted attention from researchers around the world. In this study, we selected 226 eukaryotic species from invertebrates to vertebrates to uncover evolutionary traces involved in the interplay between nucleotide and synonymous codon usages. To our knowledge, this is the first genome-wide investigation of *atg13* genes across eukaryotic organisms at the nucleotide, synonymous codon, and amino acid levels. All the target genes were separated into groups and were observed to have explicit orthologous relationships at the nucleotide and amino acid levels (**Figures 1** and **2**), implying that the *atg13* genes in either invertebrates or vertebrates share conserved functions from their common ancestors. Generally, nucleotide usages in *the atg13* gene exhibit more genetic information on its evolutionary trend than amino acid usages, strongly suggesting that synonymous codon usages serve as a genetic bridge between nucleotide usage and amino acid usages store additional evolutionary traces for the *atg13* gene.

After all, all proteins primarily derived from nucleotide organization are encoded by the corresponding transcript sequences. The previous studies reported that the *atg* genes owned different intron/exon organizations from lower organisms to higher ones (Ohsumi, 2014; Kang et al., 2018). Based on different strengths of compact genomic structures of *atg13* genes of different organisms, synonymous codon usage patterns should store additional evolutionary scenarios for different eukaryotic species. The interplay between nucleotide usage pattern and amino acid composition is considered a central platform for biological processes and probably plays an important role in the evolutionary development of the protein-based life cycle (Nicholson et al., 2018).

Specific identification of autophagy-related structures participates in clarifying autophagic activity, and clarification of autophagy-related genes plays an important role in identifying the requirements of autophagy in different animals (Kuma et al., 2017). From what we have found, the bias extents of nucleotide usages at different codon positions show that the degrees of bias at the third codon position of the *atg13* genes are more variant than those at other positions (**Figure 3**). While nucleotide usages at the third codon position reflect the existence of broad distributions of the fitness effect of synonymous mutations in the *atg13* genes during their evolution, the first and second codon positions are influenced by the conserved functions of ATG13 protein which directly dominate amino acid usages. A similar pattern can also be found during mRNA splicing where the nucleotide usages at the third codon position can be influenced by translation-independent selective pressures (Wu and Hurst, 2015). Interestingly, nuclear STAT3 fine-tunes autophagy *via* the transcriptional regulation of several autophagy-related genes (You et al., 2015), strongly suggesting that some regulators involved in various cellular signaling pathways possibly interact with *atg* genes at the transcriptional level. Among the usage bias extents for each nucleotide in the *atg13* genes (**Figure 4**), the nucleotide ‘C’ displays a relatively convergent evolution for most species, while nucleotide ‘A’ usage exhibits an obviously dispersed pattern. Although adenine is not the active component of protein-nucleotide binding, it serves as a ‘molecular handle’ which enhances binding specificity and affinity (Cai et al., 2009; Hong et al., 2009; Rogne et al., 2018). Based on the studies mentioned above, adenine is a potentially promising candidate for analyzing the evolutionary development of molecular recognition in proteins (Narunsky et al., 2020).

The strong selective forces acting on usage biases of different kinds of nucleotide contribute to synonymous codon usage bias for the *atg13* genes. Despite the divergent evolutionary pathways of the *atg13* genes across eukaryotes at synonymous codon usages (**Table S2**), some synonymous codons with conserved usage patterns retain in the *atg13* genes (**Table 1**), strongly suggesting that these synonymous codons with the conserved usage patterns benefit the *atg13* gene for sustaining the normal biological functions. Of note, the *atg13* gene and the unc-51 like autophagy activating kinase 1 (Ulk1) are the crucial substrates of the target of rapamycin complex 1 (TORC1) in autophagy (Noda, 2017), suggesting that the *atg13* gene requires one conserved sequence structure for sustaining its correct protein properties. These results also imply that synonymous codon usage patterns for the *atg13* gene are most likely to fit in the species-specific evolutionary pathway with conserved biological functions and fine-tune translation selection. Moreover, synonymous codons are not uniformly represented in the transcriptome; the observed codon bias has coevolved with tRNA abundances under selection for translation accuracy and efficiency (Ikemura, 1981; Bulmer, 1991; Drummond and Wilke, 2008; Guimaraes et al., 2020). As a result, codon usage impacts gene expression on both translation efficiency and mRNA decay process (Presnyak et al., 2015; Hanson and Coller, 2018).

To provide a reference for the *atg13* gene of *Petromyzon marinus* at the overall codon usage vs. GC3 content, we clarified the evolutionary dynamics derived from natural selection for driving the evolution of the *atg13* genes (**Figure 5**). Furthermore, RP2 plot analyses prove that natural selection plays a key role in the evolutionary pathway of the *atg13* gene, namely for the *atg13* genes of *Latimeria chalumnae, Petromyzon marinus*, and *Rhinatrema bivittatum* at synonymous codon usage patterns for Ala, Gly, Pro, Thr, and Val (**Figure 6**). This is, to our knowledge, the first report of general codon usage for *atg13* genes of different species affected by natural selection. We assume the overall codon usage pattern to be quite stable over the course of evolution since switching to a new codon usage pattern would impose a high genetic load by simultaneously modifying the selection coefficient at many synonymous positions. Since ATG13 protein plays an important role in the maintenance of cell viability under starvation conditions, *atg13* mutations, along with other transcript variants of the *atg13* gene, were defective in autophagy (Funakoshi et al., 1997; Stephan et al., 2009). Moreover, variations in synonymous codon usage are abundant across multiple levels of organization: between codons for one amino acid, between genes in a genome, and between genomes of different species (Labella et al., 2019). Here, the evolution of the *atg13* genes in different eukaryotic species represents the species-specific model at synonymous codon usages (**Figure 7**), strongly suggesting that genetic changes of synonymous codon usage in the *atg13* gene benefit the corresponding protein products with functional fit to perform the proper biological functions in specific species. Given that genetic changes in genes can alter phenotypes and even result in diseases, the synonymous codon usage bias has a strong correlation with protein biological functions (Chakraborty et al., 2017; Chakraborty et al., 2019; De Mandal et al., 2020; Malakar et al., 2020). With the ongoing analyses for the genetic signatures of *atg* genes, some members have been regarded as markers for predicting some diseases (such as cancers) (Baradaran Ghavami et al., 2019; Chen et al., 2020; Yang et al., 2020; Lai et al., 2020; Wang et al., 2020). Despite a weak pressure caused by translation selection, the selective force from natural selection plays an obvious role in GC-conservative codons in different organisms, leading to alterations of the gene structures (Carlini et al., 2001; Carlini and Stephan, 2003; Galtier et al., 2018). Based on the distributions of different nucleotide compositions with respect to the nucleotide context of the *atg13* gene (**Table 3**), it can be found that selective forces from natural selection are more significant than mutation pressure from nucleotide composition in terms of affecting the synonymous codon usage. It is worth noting that adaptive evolution caused by nucleotide compositional distribution and protein property also plays an important role in genetic diversity among genomes (Foerstner et al., 2005; Reichenberger et al., 2015).

In conclusion, based on the strong evidence of nucleotide and synonymous codon usage biases, we find the imbalance between selective forces from natural selection and mutation pressure from nucleotide composition constraint on the evolution of *atg13* genes of different species. During the formation of synonymous codon usages for the *atg13* genes, nucleotide usage patterns at the first and second codon positions are influenced by amino acid composition constraint, while nucleotide usage patterns at the third codon position are affected by translation selection. Of note, natural selection is a significant determinant of codon usage patterns in *atg13* genes across eukaryotic organisms, but mutation pressure still plays an important in the formation of codon usage of this gene in ancestry animals (such as *Petromyzon marinus*).

## Data Availability Statement

The original contributions presented in the study are included in the article/**Supplementary Material**. Further inquiries can be directed to the corresponding author.

## Author Contributions

YL, RW, and FP collected the related sequence data from NCBI website. HW, XF, and LJ analyzed and calculated the related data. ZM and XM designed and wrote the manuscript. All authors contributed to the article and approved the submitted version.

## Funding

This work was supported by the Natural Science Foundation of Gansu Province (No. 20JR5RA505), the Innovative Research Team in University (No. IRT_17R88), and the Center Universities Foundation supported by Northwest Minzu University (No. 31920200073).

## Conflict of Interest

The authors declare that the research was conducted in the absence of any commercial or financial relationships that could be construed as a potential conflict of interest.

## Publisher’s Note

All claims expressed in this article are solely those of the authors and do not necessarily represent those of their affiliated organizations, or those of the publisher, the editors and the reviewers. Any product that may be evaluated in this article, or claim that may be made by its manufacturer, is not guaranteed or endorsed by the publisher.
